# Supplementary material for: Engineered Multivalent Nanobodies Efficiently Neutralize SARS-CoV-2 Omicron Subvariants BA.1, BA.4/5, XBB.1 and BQ.1.1
Source: Vaccines (Basel). 2024 Apr 15;12(4):417. doi: 10.3390/vaccines12040417 (PMC11054741; doi:10.3390/vaccines12040417)
Supplement: Supplementary file 1 [file vaccines-12-00417-s001.zip › vaccines-2915458-supplementary.pdf]

Supplementary Materials

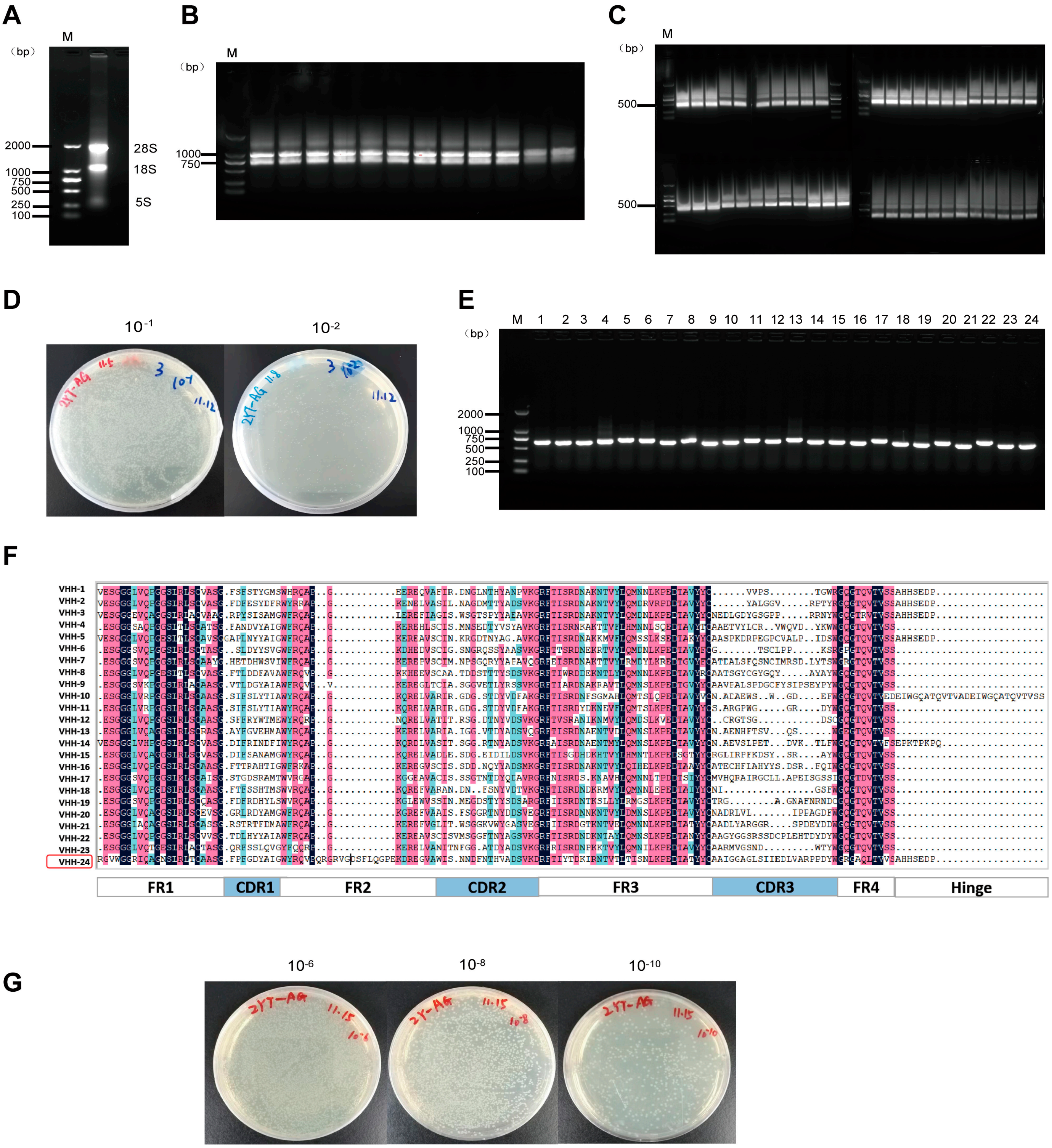

Figure S1. Construction of a VHH phage display library. (**A**) The integrity of the isolated RNA. The ratio of 28S:18S RNA was close to 2:1, which indicates that the total RNA had good integrity. (**B**,**C**) VHH genes were generated by a two-step nested PCR. (**D**) The VHH library size was measured by counting the clone numbers using the gradient dilution method. (**E**) The insertion rate of the library was estimated by randomly selecting 24 VHH colonies and performing colony-PCR. (**F**) Alignment of the amino acid sequences of 24 VHH colonies. (**G**) The size of the VHH phage display library was measured by counting the clone numbers using the gradient dilution method. M: DNA marker DL 2000.

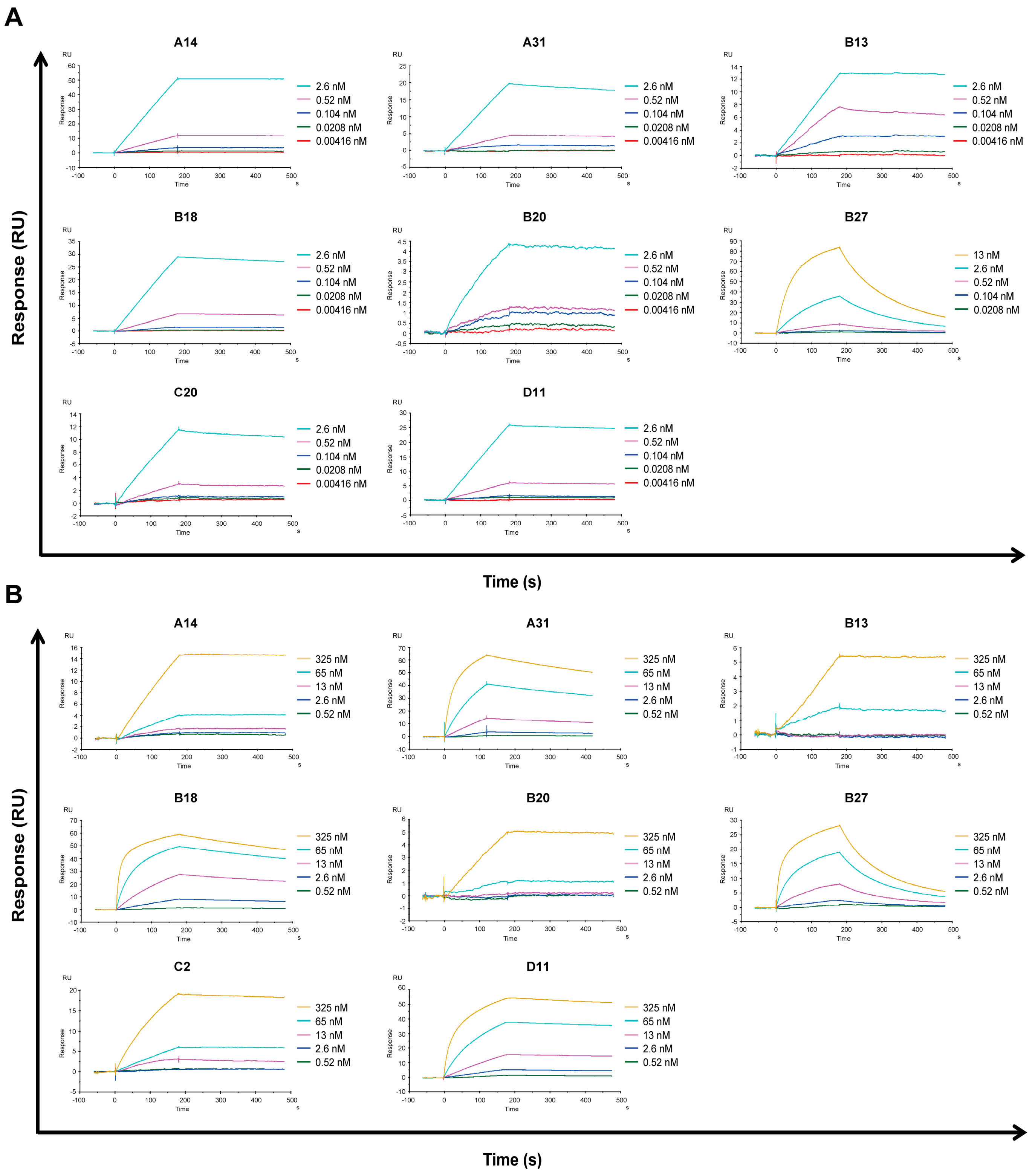

Figure S2. SPR binding kinetics of nanobodies to the RBD proteins of SARS-CoV-2 WT (**A**) and Omicron BA.1 (**B**). Serial 1:5 dilutions of nanobodies were injected and monitored by using the Biacore T200 system. The processes of association and dissociation were fitted by a 1:1 binding data fit model.

Table S1. Primer sequences for amplification of VHH genes.

Primers	Sequences	Products
AL.CH2	5′-ATGGAGAGGACGTCCTTGGGT-3′	
AL.CH2.2	5′-TTCGGGGGGAAGAYRAAGAC-3′	
1st-F	5′-GTCCTGGCTGCTCTTCTACAAGG-3′	750 bp,
1st-R	5′-GGTACGTGCTGTTGAACTGTTCC-3′	1000 bp
2nd-F1	5′-GATGTGCAGGGCCCAGCCGGCCGAGTCTGGRGGAGG-3′(Sfi I)	
2nd-R1	5′-GGACTAGTGCGGCCGCTGAGGAGACGGTGACCTGGGT-3′(Not I)	
2nd-F2	5′-TCGCGGCCCAGCCGGCCATGGCCCAGKTGCAGCTCGTGGAGTCNGGNGG -3′(Sfi I)	400 bp
2nd-R2-1	5′-CGAGTGCGGCCGCGGGGTCTTCGCTGTGGTGCG-3′(Not I)	
2nd-R2-2	5′-CGAGTGCGGCCGCTTGTGGTTTTGGTGTCTTGGG-3′(Not I)	
